# Promyelocytic Leukemia Protein Potently Restricts Human Cytomegalovirus Infection in Endothelial Cells

**DOI:** 10.3390/ijms231911931

**Published:** 2022-10-08

**Authors:** Sven Seitz, Anna Theresa Heusel, Thomas Stamminger, Myriam Scherer

**Affiliations:** Institute of Virology, Ulm University Medical Center, 89081 Ulm, Germany

**Keywords:** cytomegalovirus, HCMV, endothelial cells, HEC-LTT, PML nuclear bodies, PML, Daxx

## Abstract

PML nuclear bodies (PML-NBs) are dynamic macromolecular complexes that mediate intrinsic immunity against viruses of different families, including human cytomegalovirus (HCMV). Upon HCMV infection, PML-NBs target viral genomes entering the nucleus and restrict viral immediate–early gene expression by epigenetic silencing. Studies from several groups performed in human fibroblast cells have shown that the major PML-NB components PML, Daxx, Sp100 and ATRX contribute to this repression in a cooperative manner. Their role for HCMV restriction in endothelial cells, however, has not yet been characterized although infected endothelium is thought to play a crucial role for HCMV dissemination and development of vascular disease in vivo. Here, we use conditionally immortalized umbilical vein endothelial cells (HEC-LTT) as a cell culture model to elucidate the impact of PML-NB proteins on lytic HCMV infection. Depletion of individual PML-NB proteins by lentiviral transduction showed a particularly strong antiviral effect of PML in HEC-LTT, compared to human fibroblasts. A closer characterization of this antiviral function revealed that PML may not only effectively inhibit HCMV immediate-early gene expression but also act at later steps of the viral replication cycle. At contrast, we surprisingly noted an antiviral behavior of Daxx in complementary approaches: Depletion of Daxx resulted in decreased viral gene expression, while overexpression of Daxx promoted HCMV infection. In summary, our data demonstrate a cell type-specific effect of PML-NB components on lytic HCMV infection and suggest an important role of PML in the inhibition of HCMV dissemination through infected endothelial cells.

## 1. Introduction

Human cytomegalovirus (HCMV), also known as human herpesvirus type 5, is a widespread opportunistic pathogen that establishes a lifelong persistent infection in its host. While HCMV infection is well controlled and mostly asymptomatic in immunocompetent people, it can cause severe disease in newborns and immunocompromised patients [1,2]. In acute systemic infection of immunocompromised hosts, HCMV spreads throughout the organism via the bloodstream and infects a broad range of cell types in all major organs [3]. Vascular endothelial cells, which are located at the border between the circulation and organ tissues, are assumed to facilitate hematogenous viral dissemination through the transmission of virus to neighboring cells or adhering leukocytes, release of virus into the blood stream and detachment of infected endothelial cells [4,5]. HCMV-infected endothelium has also been implicated in the development of vascular diseases, e.g., atherosclerosis, and may act as a viral reservoir for HCMV persistence [6,7].

The first successful infection of endothelial cells in vitro was performed in primary umbilical vein endothelial cells (HUVEC), which have become the standard endothelial cell culture model for HCMV [5,8]. The use of HUVEC for standardized and long-term experiments, however, is hampered by the fact that they possess a limited life span and show donor variability. Recently, a conditionally immortalized, HUVEC-based cell line (HEC-LTT) was developed and subsequently demonstrated to support the full HCMV replication cycle leading to the release of infectious progeny virus [9,10]. HEC-LTT have been generated by introduction of inducible expression cassettes for simian virus 40 large T-antigen (SV40-TAg) and human telomerase catalytic subunit (hTERT), which are transcribed in the presence of doxycycline (Dox) and lead to unlimited cell proliferation. Upon removal of Dox, HEC-LTT stop to proliferate and can be cultured in a growth arrested state resembling endothelial cells in vivo [9,10]. Therefore, HEC-LTT appear as useful tool to analyze different events during HCMV replication in infected endothelium.

Upon infection of a host cell, viruses are recognized and inhibited by an array of cellular defense mechanisms. The intrinsic immune response forms a first line of protection against viral infections, as it is conferred by constitutively expressed and active proteins called restriction factors [11]. Several restriction factors including PML, Sp100, Daxx, ATRX and MORC3, that have been shown to inhibit human cytomegalovirus replication, localize to cellular structures known as PML nuclear bodies (PML-NBs) or nuclear domain 10 (ND10) [12,13,14,15]. These dynamic and heterogeneous protein complexes appear as 1–30 nuclear dots, depending on cell type and condition, and are formed by the key component PML [16]. The numerous isoforms of PML are subject to covalent modification with small ubiquitin-like modifier (SUMO) proteins, which drives the recruitment of client proteins and is therefore a prerequisite for PML-NB formation [17]. Due to the high number of proteins that can be recruited, PML-NBs have been implicated in diverse cellular processes, such as transcriptional regulation, DNA damage response, survival and apoptosis as well as antiviral defense. Upon HCMV infection, PML-NBs sense and entrap viral genomes entering the nucleus and induce epigenetic silencing of the viral DNA by recruiting chromatin-modifying enzymes [18,19]. This restrictive activity allows PML-NBs to block one of the first steps in the HCMV life cycle. However, it is antagonized by the regulatory HCMV proteins pp71 and IE1. The tegument-delivered protein pp71 is imported into the nucleus immediately upon infection, where it leads to the displacement of ATRX from PML-NBs, followed by proteasomal degradation of Daxx [14,20]. As this facilitates viral gene expression, the immediate–early protein 1 (IE1) can be expressed, which inhibits the SUMOylation of PML and Sp100 [21,22,23]. Due to this activity, IE1 induces a complete disruption of PML-NBs within the first hours of infection thereby enabling the onset of lytic HCMV replication.

In this study, we use the HEC-LTT model system to analyze the effect of PML-NBs on lytic HCMV infection in endothelial cells, as the above-described findings are limited to studies in human fibroblasts. Depletion of individual PML-NB proteins by lentiviral transduction revealed an exceptionally strong antiviral effect of PML in HEC-LTT cells, whereas Daxx was found to promote HCMV infection. Thus, our data provide evidence that the antiviral function and efficiency of PML-NB proteins against a certain virus varies considerably in different cell types, with PML emerging as an effective restriction factor for HCMV in infected endothelium.

## 2. Results

### 2.1. PML-NB Protein Expression in Endothelial Cells and Fibroblasts

**Figure 1:**
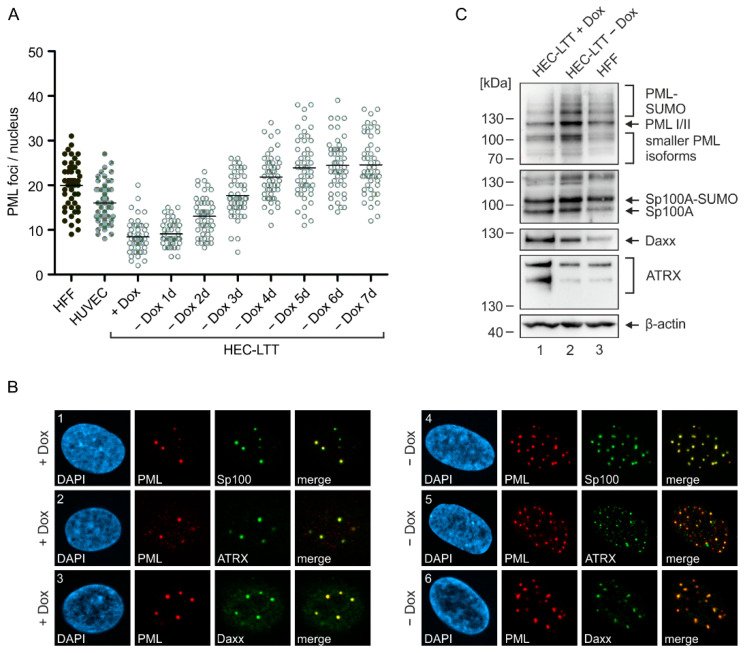
Characterization of PML-NBs in HEC-LTT. (**A**) Mean number of PML-NBs in the nuclei of HEC-LTT compared to HUVEC and HFF cells shown in a scatter plot. HEC-LTT cells were cultured in the presence of doxycycline (+Dox) or were deprived of doxycycline for one to seven days to induce growth arrest. HEC-LTT, HUVEC, and HFF cells were fixed and subjected to indirect immunofluorescence analysis of endogenous PML. Subsequent quantification of PML foci was performed in 50 cells per sample using maximum intensity projections of z-series images. (**B**) Localization of PML-NB proteins in HEC-LTT. HEC-LTT were cultured in presence (+Dox) or in absence (−Dox) of doxycycline for five days, before they were harvested and subjected to immunofluorescence analysis. Antibodies against PML and either Sp100, ATRX or Daxx were used. Representative images for the co-localization of PML-NB proteins are shown, which was analyzed in >50 cells per sample. (**C**) Western Blot analysis of PML-NB proteins in HEC-LTT and HFF cells. HEC-LTT were cultured in presence (+Dox) or absence (−Dox) of doxycycline for five days. HEC-LTT and HFF samples were harvested for Western blot analysis of PML, Sp100, Daxx, ATRX and β-actin as loading control. One representative Western blot experiment of ≥2 is shown.

While the impact of PML-NBs on lytic HCMV infection has been extensively studied in fibroblasts, their role in endothelial cells, which promote the spread of HCMV in vivo [4,5], remains unknown. To address this issue, we made use of conditionally immortalized endothelial cells (HEC-LTT) that stop unlimited proliferation when cultured in absence of doxycycline. Since the number of PML-NBs has been reported to vary in different cell types and also cell cycle states [16], we first quantified PML-NBs in both proliferating as well as growth arrested HEC-LTT. For this, HEC-LTT were deprived of doxycycline for up to seven days and subjected to immunofluorescence staining of endogenous PML, followed by quantification of PML foci in maximum intensity projection images. Compared to primary HUVEC, from which HEC-LTT originate, we detected a lower number of PML-NBs in proliferating HEC-LTT (Figure 1A, +Dox). However, PML foci clearly increased after removal of doxycycline (Figure 1A, −Dox). Since the number of PML-NBs in HEC-LTT reached a steady-state level at five days of doxycycline withdrawal, which was only slightly higher than the mean foci number in primary human fibroblasts (HFF), all following infection experiments were performed under these conditions.

To analyze the localization of the other main PML-NB components Sp100, Daxx, and ATRX, we performed a co-staining with PML in proliferating and growth arrested HEC-LTT. We found that all three proteins are expressed in the cell nucleus and are recruited to PML foci (Figure 1B), as described in other cell types such as HFF [13,24]. Again, doxycycline treatment affected the number of PML-NBs per nucleus; however, no difference in the co-localization of PML-NB proteins was observed in proliferating (Figure 1B, panel 1–3) and growth arrested (Figure 1B, panel 4–6) HEC-LTT. Interestingly, Western blot analysis revealed an upregulation of the various PML and Sp100 isoforms after doxycycline removal, whereas Daxx and ATRX were downregulated (Figure 1C, compare lane 1 and 2). Compared to HFF cells, growth arrested HEC-LTT (HEC-LTT − Dox) displayed slightly higher abundances of PML, Sp100 and Daxx, while comparable levels of ATRX were detected (Figure 1C, compare lane 2 and 3). Overall, however, our data show a similar expression and localization of all major PML-NB proteins in endothelial cells and fibroblasts.

### 2.2. Disruption of PML-NBs in Endothelial Cells during HCMV Infection

**Figure 2:**
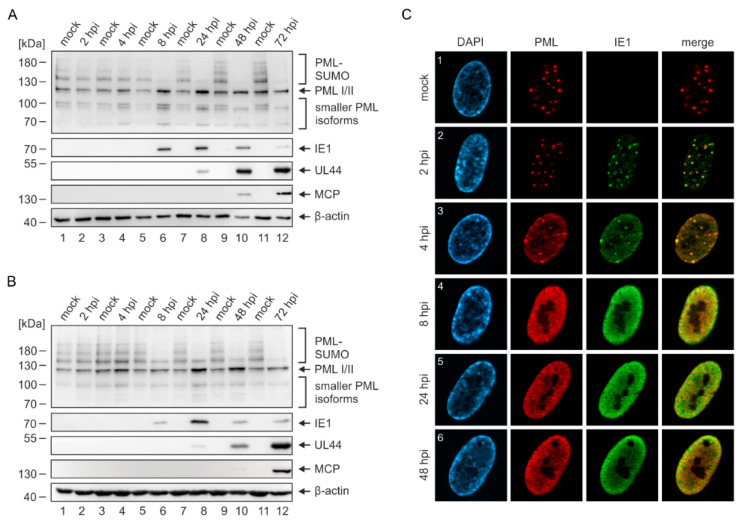
Disruption of PML-NBs in HCMV-infected HEC-LTT. (**A**,**B**) Western Blot analysis of PML SUMOylation during HCMV infection. Growth-arrested HEC-LTT (**A**) and HFF (**B**) were infected with HCMV strain TB40/E at a MOI of 5. At indicated hours post infection (hpi), cells were harvested and subjected to Western blot analysis of endogenous PML as well as immediate–early (IE1), early (UL44), and late (MCP) phase viral proteins. β-actin was included as internal control. (**C**) Intracellular localization of PML and IE1 in infected HEC-LTT. Growth-arrested HEC-LTT were infected at a MOI of 5 with TB40/E. At different time points after infection, cells were fixed and stained by indirect immunofluorescence for PML and IE1. The depicted protein distributions were observed in >90% of analyzed cells (≥100 cells per sample).

Upon HCMV infection of HFF cells, the IE1 protein leads to a rapid loss of SUMOylated PML and to a disruption of PML-NBs [21,25]. To investigate whether this can likewise be observed in HEC-LTT, we infected HEC-LTT and HFF in parallel with the endotheliotropic HCMV strain TB40/E at a high multiplicity. At different times after infection, cells were harvested for Western blot analysis of PML as well as viral immediate–early (IE1), early (UL44), and late (MCP) proteins as a control for successful infection. Beginning at 8 h post infection (hpi), when IE1 was expressed in sufficient amounts, we detected a depletion of SUMOylated PML and a concomitant increase of unmodified PML variants in both HEC-LTT (Figure 2A) and HFF (Figure 2B). Immunofluorescence analysis of infected HEC-LTT revealed that IE1 initially co-localizes with PML foci (Figure 2C, panels 2 and 3), before IE1 and PML are redistributed into a diffuse nuclear pattern (Figure 2C, panels 4–6). This disruption of PML-NBs occurred in parallel to the loss of SUMOylated PML (Figure 2A,C), which is in line with previous results obtained in HFF cells [23]. Therefore, we conclude that HCMV modulates PML-NBs in the same way in endothelial cells and fibroblasts.

### 2.3. Generation of Endothelial Cells with a Stable Knockdown of PML-NB Proteins

**Figure 3:**
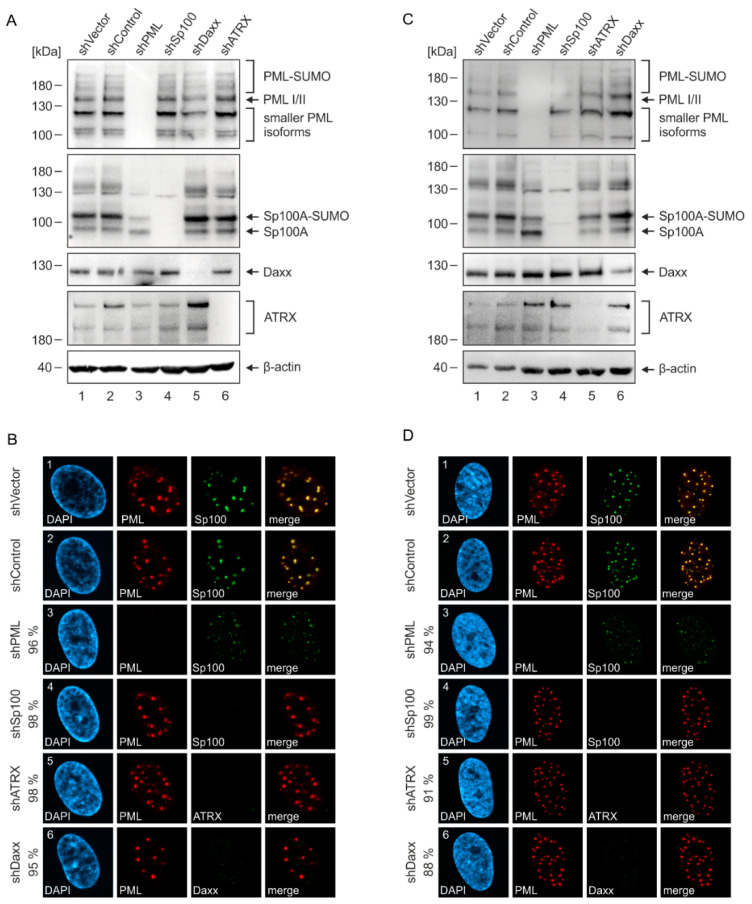
Knockdown of individual PML-NB proteins in HEC-LTT and HFF cells. HEC-LTT (**A**,**B**) and HFF (**C**,**D**) were transduced with lentiviruses generated from an empty vector (shVector), a vector containing a non-functional shRNA (shControl) or vectors encoding shRNAs directed against PML, Sp100, Daxx and ATRX (shPML, shSp100, shATRX, shDaxx). Knockdown of the individual PML-NB proteins was confirmed by Western Blot (**A**,**C**) and immunofluorescence (**B**,**D**) analysis using indicated antibodies. The knockdown efficiency of the respective proteins was determined by analysis of >100 cells in immunofluorescence images and is indicated for HEC-LTT (**B**) and HFF (**D**) cells.

Having shown that HEC-LTT express all major PML-NB proteins (Figure 1B,C), we next depleted individual PML-NB components in order to characterize their impact on HCMV replication. For this purpose, HEC-LTT were transduced with lentiviruses expressing shRNAs directed against PML, Sp100, Daxx or ATRX. Lentiviruses containing either an empty vector (shVector) or a control shRNA (shControl) were used to establish control HEC-LTT. Subsequent selection of puromycin-resistant cells resulted in an efficient knockdown of the individual NB proteins, as confirmed by Western blotting (Figure 3A) as well as immunofluorescence analysis (Figure 3B). In order to directly compare the effects of PML-NB protein knockdown in endothelial cells and fibroblasts, the same set of knockdown cells was generated using HFF (Figure 3C,D). Consistent with published data on HFF, we observed that depletion of PML, but not of Daxx or ATRX, has substantial effects on the various Sp100 bands and leads to a shift from SUMOylated Sp100A towards the non-SUMOylated form (Figure 3C) [23,26]. Immunofluorescence analysis revealed that only few and less intense Sp100 foci are present in PML-knockdown HFF (Figure 3D, panel 3). In contrast, depletion of Sp100, Daxx or ATRX did not affect the localization of PML, reflecting its role as key organizer of PML-NBs (Figure 3D, panel 4–6). These observations were also made in HEC-LTT (Figure 3A,B) and, again, point towards a similar regulation of PML-NBs in fibroblasts and endothelial cells.

### 2.4. Enhanced Initiation of HCMV Gene Expression in PML- but Not Daxx-Depleted HEC-LTT

**Figure 4:**
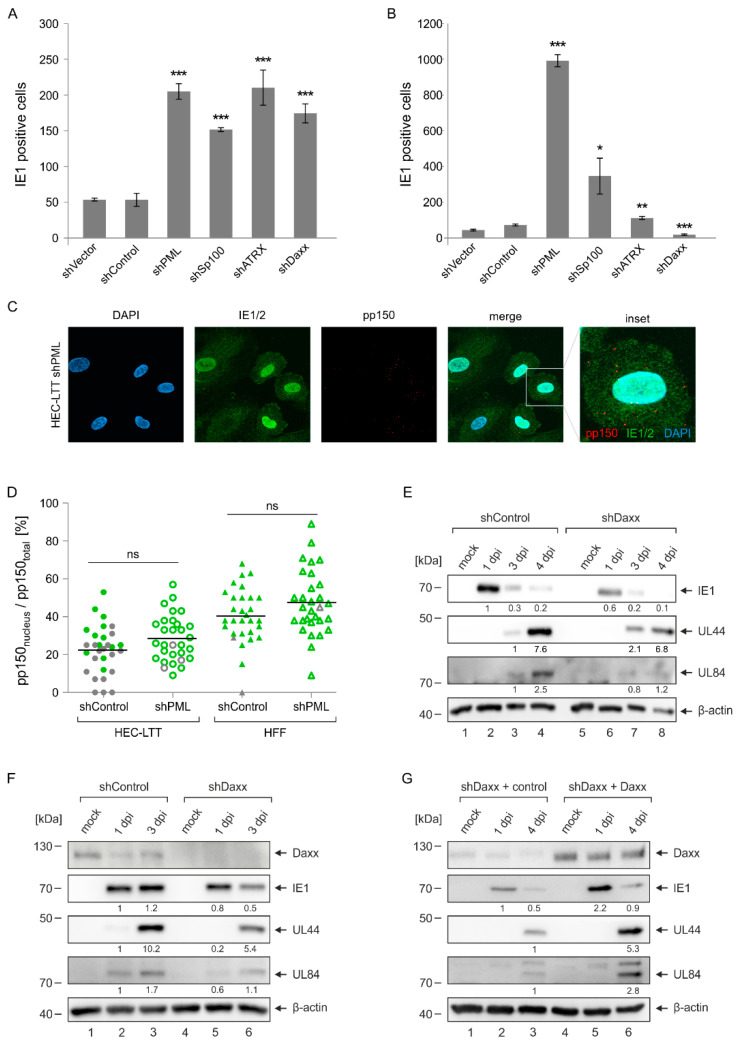
Analysis of HCMV IE gene expression in HEC-LTT and HFF with a knockdown of PML-NB proteins. (**A**,**B**) Effect of PML-NB protein knockdown on initiation of HCMV IE1 gene expression. HFF (**A**) and growth-arrested HEC-LTT (**B**) with a knockdown of PML-NB proteins (shPML, shSp100, shDaxx, shATRX) as well as control cells (shVector, shControl) were infected with 50 immediate–early units of TB40/E. At 24 hpi, the number of IE-expressing cells was determined by fluorescence microscopy using an IE1-specific antibody. Values are derived from triplicate samples and represent mean values ± SD. The results are representative for two independent experiments performed in triplicates. Datasets were analyzed by one-way-ANOVA to test for significant differences between the various cell populations. Post-hoc analysis was conducted to compare the individual datasets with shControl cells, which were used as control cells in all following experiments. Asterisks indicate statistically significant differences. ***, *p* < 0.001; **, *p* < 0.01; *, *p* < 0.05. (**C**,**D**) Effect of PML knockdown on nuclear translocation of HCMV particles. PML-knockdown and control HEC-LTT (growth-arrested) and HFF were infected with freshly harvested TB40/E. After 6 h, cells were fixed for immunofluorescence staining of the viral tegument protein pp150 and of viral IE1/IE2. An exemplary image of HEC-LTT shPML is shown (**C**). To determine the nuclear translocation efficiency of HCMV particles, pp150 signals at the nucleus were counted in 30 cells derived from three independent experiments and are shown as percentage of total intracellular pp150 signals. IE1/IE2 expression in the single cells is indicated by green coloring, grey color is used for cells in which no IE gene expression was initiated. Student’s *t*-test was performed for statistical analysis. ns, not significant (**D**). (**E**) Effect of Daxx depletion on HCMV infection in HEC-LTT. Growth-arrested control (shControl) and Daxx-knockdown (shDaxx) HEC-LTT were infected with TB40/E at a MOI of 3. At indicated times, the cells were subjected to Western Blot analysis of HCMV immediate–early (IE1) and early (UL44, UL84) proteins. β-actin was included as internal control. Levels of viral proteins were quantified via densitometric analyses using the Fusion Software (Vilber Lourmat GmbH). Protein levels were normalized to the internal control (β-actin) and are given as fold change. (**F**) Effect of Daxx depletion on HCMV infection in primary HUVEC. HUVEC were incubated with control lentiviruses (shControl) or lentiviruses encoding a shRNA against Daxx (shDaxx). Five days after transduction, the cells were infected with TB40/E at a MOI of 3. At indicated times after HCMV infection, cells were subjected to Western Blot analysis of Daxx as well as viral immediate–early (IE1) and early (UL44, UL84) proteins. β-actin was included as internal control. Levels of viral proteins were quantified as described in (**E**). (**G**) Effect of Daxx reintroduction on HCMV infection in HEC-LTT. Daxx-depleted HEC-LTT were transduced with control lentiviruses (shDaxx + control) or lentiviruses expressing Flag-tagged Daxx (shDaxx + Daxx) and selected with blasticidin, before they were mock infected or infected with TB40/E at a MOI of 3. At indicated times after infection, cells were subjected to Western Blot analysis of Daxx as well as viral immediate–early (IE1) and early (UL44, UL84) proteins. β-actin was included as internal control. Levels of viral proteins were quantified as described in (**E**).

Since PML-NBs are known to repress the transcription of incoming HCMV genomes, we next investigated the effect of NB protein knockdown on HCMV immediate–early (IE) gene expression. For this, the newly generated HEC-LTT and HFF cells were infected with a low infectious dose of HCMV, followed by determination of IE1-positive cells at 24 hpi via immunostaining. We found that depletion of the major PML-NB proteins in HFF cells consistently resulted in a three- to fourfold higher number of IE1-expressing cells, which is in line with already published results (Figure 4A) [13,14]. Surprisingly, we detected a considerably stronger effect of PML depletion in HEC-LTT, as approximately 20 times more cells displayed viral gene expression when compared to control cells (Figure 4B). Knockdown of Sp100 also resulted in a clear increase of IE gene expression, whereas ATRX-depleted HEC-LTT showed an only slight increase and Daxx-depleted cells even a decrease in viral IE gene expression.

To exclude that the strong antiviral effect of PML in HEC-LTT is due to an inhibition of viral entry steps that precede IE gene expression, we next analyzed the transport of virions to the cell nucleus. For this, PML-knockdown and control cells were incubated with freshly harvested HCMV supernatant, followed by staining of the tegument protein pp150 as a marker for HCMV particles and IE1/2 as a marker for viral gene expression as previously described by Sinzger et al. [27] (Figure 4C). Comparison of pp150 signals that have reached the cell nucleus revealed a slight but not significant increase in nuclear localization efficiency in PML-depleted HEC-LTT and likewise in PML-depleted HFF cells (Figure 4D). Despite the similar transport efficiency of virions to the nucleus of HEC-LTT cells, a higher number of IE1-positive cells was detected in PML-knockdown compared to control HEC-LTT, as indicated by green coloring in Figure 4D. This finding suggests that PML does not affect viral entry and transport in HEC-LTT but acts as a strong repressor of viral IE gene expression.

In contrast to PML, the Daxx protein appeared to have a rather pro-viral effect in HEC-LTT (Figure 4B). To corroborate this finding, we examined viral protein accumulation at different times after HCMV infection by Western blot analysis. As shown in Figure 4E, we detected lower abundances of viral IE and E proteins in Daxx-depleted HEC-LTT compared to control cells. Equivalent results were obtained in primary HUVEC that, due to their limited life span, were incubated with Daxx-knockdown or control lentiviruses and subsequently superinfected with HCMV. Since a reduced accumulation of HCMV proteins was also observed in Daxx-depleted HUVEC (Figure 4F), we conclude that the antiviral function of Daxx is not specific to HEC-LTT but represents a general feature of endothelial cells. Finally, we examined whether re-introduction of Daxx into Daxx-knockdown HEC-LTT can promote HCMV infection. Previous studies of our group have demonstrated that overexpression of a protein without introducing mutations can serve as successful means to overcome siRNA-mediated gene silencing and restore protein expression [12,28]. Therefore, Daxx-knockdown HEC-LTT were transduced with lentiviruses coding for Flag-tagged Daxx or control lentiviruses, followed by selection of puromycin- and blasticidin-resistant cells. Analysis of HCMV gene expression showed that, contrary to previous observations in HFF cells, Daxx indeed stimulates viral IE and E gene expression in HEC-LTT (Figure 4G) [12]. Taken together, this evidence suggests that function and efficiency of PML-NB proteins varies in different cell types, with PML appearing as effective restriction factor in endothelial cells.

### 2.5. Multiplicity-Independent Inhibition of HCMV Infection by PML

**Figure 5:**
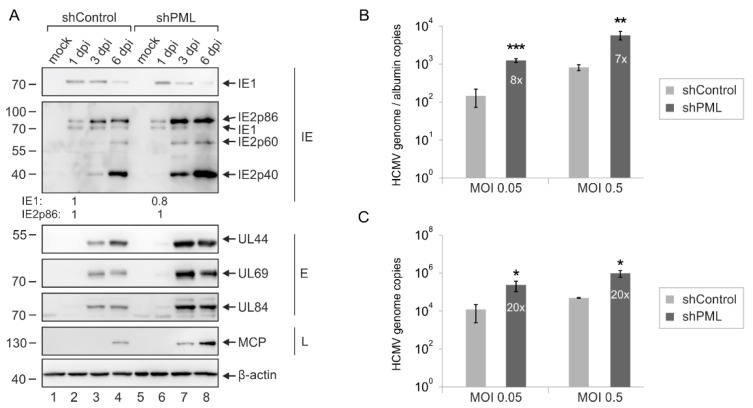
MOI-independent inhibition of HCMV replication by PML in HEC-LTT. (**A**) Analysis of HCMV gene expression in PML-knockdown (shPML) HEC-LTT compared to control cells (shControl). Growth-arrested HEC-LTT were infected with TB40/E at a MOI of 1 and harvested at different times after infection. Western blot analysis was conducted to assess the abundances of viral immediate–early (IE), early (E), and late (L) proteins as indicated. β-actin was included as internal control. Levels of viral IE proteins at 24 hpi were quantified via densitometric analysis using the Fusion Software (Vilber Lourmat GmbH) and normalized to the internal control (β-actin). The mean densitometric values of three Western blots are given as fold-changes. (**B**) Quantification of HCMV DNA replication in PML-knockdown HEC-LTT compared to control cells. Growth-arrested PML-knockdown (shPML) and control (shControl) HEC-LTT were infected with TB40/E at a MOI of 0.05 or 0.5. At 5 dpi, intracellular DNA was extracted, and viral genome equivalents were determined via IE1-specific qPCR in relation to cellular albumin copy numbers. Values are derived from biological triplicates and represent mean values ± SD. Data sets were analyzed using unpaired Student’s *t*-tests. Asterisks indicate statistically significant differences. **, *p* < 0.01; ***, *p* < 0.001. (**C**) Quantification of virus progeny release from PML-knockdown HEC-LTT compared to control cells. Growth-arrested PML-knockdown and control HEC-LTT were infected with TB40/E at a MOI of 0.05 or 0.5. At 5 dpi, viral supernatants were harvested and subjected to protease K treatment followed by IE1-specific qPCR. Standard deviations of three biological replicates are shown. P-values were calculated using unpaired Student’s *t*-tests and are indicated by asterisks. *, *p* < 0.5.

In order to further characterize the antiviral role of PML in HEC-LTT, we determined the effect of PML depletion on expression of viral early and late genes. For this purpose, HEC-LTT were infected with TB40/E at a MOI of 1 and subjected to Western Blot analysis of different immediate–early (IE1, IE2p86), early (UL44, UL69, UL84) and late (MCP, IE2p40, IE2p60) proteins. As shown in Figure 5A, similar levels of IE proteins were detected at 1 dpi in control and PML knockdown cells. This is in line with the previous observation that restriction factors can be saturated by high virus doses and that antiviral effects of PML-NBs proteins on HCMV IE gene expression are no longer detectable at MOIs ≥ 0.5 [23,28]. Nevertheless, considerably higher abundances of HCMV proteins were present at later stages of infection (≥48 hpi) in PML-depleted HEC-LTT compared to control cells (Figure 5A). In accordance, we detected enhanced viral DNA synthesis, as determined by DNA isolation from infected cells and qPCR measurement (Figure 5B). Analysis of viral genomes in the cellular supernatants furthermore showed that PML depletion also resulted in an increased release of HCMV particles from HEC-LTT (Figure 5C). Interestingly, comparable antiviral effects were observed after infection with different viral loads (MOI 0.05 and MOI 0.5). Taken together, these findings indicate that PML may not only affect IE gene expression but have an additional antiviral function during later times of viral replication, thus supporting its role as an effective inhibitor of HCMV in endothelial cells.

## 3. Discussion

PML-NBs have been shown to mediate an intrinsic defense against a variety of DNA and RNA viruses. The extent and mechanisms, however, by which individual PML-NB proteins and their isoforms inhibit different viruses are highly variable [29]. Some viruses even exploit specific PML-NB proteins for their own benefit [30]. The impact of PML-NBs on lytic HCMV infection has been characterized by numerous studies in primary human fibroblasts, which revealed an inhibitory effect of the main NB components PML, Sp100, Daxx and ATRX on viral immediate–early (IE) gene expression [12,13,14,15]. Here, by using HEC-LTT with a knockdown of PML-NB proteins (Figure 3), we show that PML has a particularly strong antiviral effect in endothelial cells, whereas Daxx shows an unexpected proviral function (Figure 4). To exclude that this different behavior of PML-NB proteins in HEC-LTT is caused by variations in experimental procedures, we also analyzed the effect of PML-NB depletion in primary fibroblasts (HFF) that were generated in parallel and displayed similar effects as described in literature (Figure 4) [12,13,14,15].

Such differences in the functionality of proteins may result from cell-type specific localization or expression. Immunofluorescence analysis of the subcellular distribution of PML-NB proteins in HEC-LTT, however, revealed the typical formation of PML foci inside the nucleus that co-localize with Sp100, Daxx and ATRX (Figure 1). Quantification of PML foci showed that dividing HEC-LTT, cultured in the presence of doxycycline, have a quite low number of PML-NBs. This may be due to their high proliferation rate, since PML-NBs dissolve during mitosis and their presence is therefore cell cycle dependent [31]. After doxycycline removal, however, number of PML bodies increased and reached a steady state level of about 24 PML-NBs comparable to the mean number of 20 PML-NBs determined in HFF cells (Figure 1). Additional characterization of PML-NB proteins by Western blotting showed similar expression patterns of the various isoforms in HEC-LTT and HFF. At this point, however, a differential expression of PML isoforms cannot fully be excluded due to the numerous bands that arise from non-, mono-, and poly-SUMOylated forms of the at least seven PML isoforms. Thus, the depletion of single PML isoforms and evaluation of their contribution to HCMV restriction in both endothelial cells and fibroblasts may be a worthy subject for future investigations.

The repression of HCMV IE gene expression by PML-NBs involves the sensing and entrapment of viral genomes that enter the nucleus, followed by induction of a transcriptionally inactive chromatin state [19,32]. Although the association of PML-NBs with viral genomes has already been observed more than 20 years ago, the mechanism of genome sensing remains poorly understood [33,34]. Live-cell microscopy imaging of HSV-1-infected cells has shown that PML-NB components relocalize to sites of infecting HSV-1 genomes (visualized via the viral DNA binding protein ICP4), where de novo PML-NB-like foci are formed [35]. For HCMV infections, however, such a dynamic behavior of PML-NBs has not been observed and sensing appears to be rather inefficient, when more than one genome is present in a cell nucleus [19]. Therefore, a possible explanation for the enhanced antiviral activity of PML in endothelial cells may be a higher sensing / entrapment efficiency of viral genomes. As single cells show a high variation in the number of virus particles that reach the nucleus (Figure 4) and also in the time in which viral DNA (vDNA) enters the nucleus, investigation of this issue will require technically challenging real-time analysis of the PML-vDNA association. As an alternative hypothesis, PML may induce a more effective repression of viral chromatin by recruiting a different set or amount of chromatin modifying enzymes. The mechanism of PML-based transcriptional repression has not been fully elucidated, but it has been suggested that PML can exert its antiviral function independent of Sp100 or Daxx and may act via a direct interaction and recruitment histone deacetylases (HDACs) to viral genomes [12,36]. Therefore, future experiments e.g., mass spectrometry-based approaches aiming at a comparison of PML-interacting proteins in fibroblasts and endothelial cells may not only further clarify the PML-based inhibition of viral gene expression but also unravel cell-type specific differences.

Interestingly, PML depletion from HEC-LTT not only enhanced viral IE gene expression after low multiplicity infection but also promoted HCMV infection with higher virus doses, which usually leads to a saturation of restriction factors so that antiviral effects are no longer detectable. In previous studies, it was observed that HCMV infection at a multiplicity of 0.5 (or higher) already leads to equal IE1 expression in control and knockdown HFF [23,28]. This is in accordance with Western blot analysis of PML-knockdown and control HEC-LTT, which revealed a comparable accumulation of IE1 and IE2 in the first 24 h upon HCMV infection (Figure 5). Nonetheless, we noted augmented levels of viral early and late proteins in PML-depleted HEC-LTT during later phases of infection. Quantification of viral DNA by qPCR analysis corroborated this result, as depletion of PML resulted in a comparable increase of intracellular as well as released viral DNA after infection with different virus doses (Figure 5). A similar phenomenon was previously described in Sp100-depleted HFF cells suggesting that PML-NB proteins can exert a dual antiviral role by acting on both immediate–early and late stages of HCMV infection [23,28].

In contrast to the knockdown of PML, Sp100 depletion had a similar effect on HCMV IE gene expression in HEC-LTT compared to HFF cells, ATRX depletion showed a weaker and Daxx depletion even a contrary effect. This proviral behavior of Daxx in endothelial cells was unexpected, since Daxx has been described as a broad-spectrum restriction factor limiting the replication of different DNA viruses, such as herpes- and adenoviruses, of RNA viruses including HIV and, according to very recent findings, SARS-CoV-2 [37,38]. To our knowledge, only for papillomaviruses a positive role of Daxx on early gene expression could be detected [39]. To substantiate the finding of a proviral function of Daxx in endothelial cells, we re-introduced Daxx in shDaxx-expressing HEC-LTT by lentiviral transduction. This complementary approach revealed a clearly positive effect of Daxx overexpression on HCMV gene expression (Figure 4). The underlying mechanism awaits further clarification. However, since a recent publication described that Daxx can also act as a potent protein-folding enabler that prevents and reverses protein aggregation, one may speculate that endothelial cells may require this activity of Daxx to support HCMV replication [40].

## 4. Materials and Methods

### 4.1. Cells and Viruses

HEC-LTT cells were kindly provided by the group of Christian Sinzger (Ulm, Germany) and are described in [10], where they are named “HUVEC uni-Tag” and “bi-hTert”. HEC-LTT were used from passage 40 to 140 and were maintained in endothelial cell growth medium (EGM, Growth medium kit by PromoCell, Heidelberg, Germany) supplemented with 2 μg/mL doxycycline (Sigma-Aldrich, St. Louis, MO, USA). For passaging, the cells were washed twice with solution A (137 mM NaCl, 5.4 mM KCl, 4.2 mM NaHCO_3_, 5 mM D-Glucose) before they were treated with 0.05% trypsin-EDTA solution (Gibco, Life Technologies, Carlsbad, CA, USA) and seeded in cell culture vessels coated with 0.1% gelatin (Sigma-Aldrich, St. Louis, MO, USA). All experiments were performed 5 days after withdrawal of doxycycline, unless otherwise indicated. Human foreskin fibroblast (HFF) cells were cultured in Eagle´s minimal essential medium (MEM) containing 7% fetal calf serum (FCS, Sigma-Aldrich, St. Louis, MO, USA), glutamax (Gibco, Life Technologies, Carlsbad, CA, USA), and penicillin-streptomycin (Sigma-Aldrich, St. Louis, MO, USA). Infection of HEC-LTT and HFF with the HCMV strain TB40E-Bac4 was performed by incubation of cells with infectious supernatant, which was removed after 90 min and replaced with fresh cell culture medium [27]. Prior to infection, HEC-LTT were treated with MEM for 30 min. HEK293T cells were cultivated in Dulbecco´s minimal essential medium (DMEM) that was supplemented with 10% FCS, glutamax, and penicillin-streptomycin.

For titration of TB40/E supernatants, HFF or HEC-LTT were infected with serial dilutions of virus supernatants. 24 h after infection, cells were fixed and stained for IE1. Subsequently, the number of IE1-positive cells was determined, and viral titers were calculated as immediate–early units/mL.

### 4.2. Lentiviral Gene Transfer

In order to establish HEC-LTT and HFF with a stable knockdown of PML-NB proteins, lentiviral gene transfer was performed using the Lenti-X™ shRNA Expression System (Clontech, Takara Holdings, Kyōto, Japan). For this, the nucleotide sequences of short hairpin RNAs (shRNAs) targeting PML, Sp100, Daxx and ATRX were inserted into pLVX-shRNA1 via BamHI and XhoI as described previously [41,42]. Furthermore, an empty vector pLVX-shRNA1 (shVector) and a vector, in that a non-functional half-side shRNA (shControl) was inserted, were used as control. The respective small interfering RNA (siRNA) target sequences are listed in Table 1. For reintroduction of Flag-Daxx, the Flag-Daxx fusion sequence was cloned in the pLenti6/V5-D-TOPO vector (Invitrogen, Life Technologies, Carlsbad, CA, USA) as described previously [12]. As control, a pLenti6.4/R4R2/V5-Dest-based empty vector (Invitrogen, Life Technologies, Carlsbad, CA, USA) was used. To generate lentiviral supernatants, HEK293T cells were seeded in 10 cm dishes at a density of 5 × 10^6^ cells/dish and, one day later, were transfected with pLVX-shRNA1-based vectors together with packaging plasmids pLP1, pLP2, and pLP/VSV-G using the Lipofectamine 2000 reagent (Invitrogen, Life Technologies, Carlsbad, CA, USA). Viral supernatants were harvested 48 h after transfection and filtered through a 0.45-µm filter before they were either directly used for transduction or stored at −80 °C. Transduction of HFF and HEC-LTT was performed by incubating the cells with supernatant in the presence of 7.5 µg/mL polybrene for 24 h. Finally, stably transduced cells were selected and cultivated by adding puromycin (Invivogen, San Diego, CA, USA) in a concentration of 5 μg/mL to the cell-specific medium. Selection of cells after transduction with encoding lentiviruses encoding Flag-Daxx or control lentiviruses was performed by adding blasticidin (Invivogen, San Diego, CA, USA) at a concentration of 2 µg/mL to the cell culture medium.

### 4.3. Western Blotting

HFF and HEC-LTT were pelleted and subsequently lysed in a sodium dodecyl sulfate-polyacrylamide gel electrophoresis (SDS-PAGE) loading buffer at 95 °C for 10 min and sonicated for 1 min. SDS-containing 8% polyacrylamide gels were used to separate proteins. The proteins were transferred to PVDF membranes (Bio-Rad Laboratories, Hercules, CA, USA) and detected by chemoluminescence using a FUSION FX7 imaging system (Vilber Lourmat GmbH, Eberhardzell, Germany).

### 4.4. Indirect Immunofluorescence

For indirect immunofluorescence, HFF and HEC-LTT were seeded at a density of 3 × 10^5^ cells/well on coverslips placed in 6-well plates. Before seeding HEC-LTT, the coverslips were coated with 0.1% gelatin (Sigma-Aldrich, St. Louis, MO, USA). At indicated times after infection, the cells were washed with PBS and fixed with a 4% paraformaldehyde solution for 10 min. After washing twice with PBS, the cells were permeabilized at 4 °C for 10 min using 0.1% Triton X-100 in PBS. Cells were washed again with PBS for four times and incubated in PBS at room temperature for 5 min. Primary antibodies were diluted in 1% FCS in PBS and applied to the coverslips for 30 min at 37 °C. Before adding the corresponding fluorescence-coupled secondary antibodies, cells were washed with PBS for three times. Coverslips were mounted onto microscope slides with DAPI-containing Vectashield mounting medium (Vector Laboratories, Newark, CA, USA). Cells were analyzed using a Zeiss Axio Observer Z1 with an Apotome.2 (Zeiss, Oberkochen, Germany). Pictures were processed with ZEN2 software and compiled using CorelDraw 2018.

### 4.5. Nuclear Localization Assay

Analysis of the nuclear localization efficiency of HCMV was performed using a slightly modified protocol as described by Sinzger and colleagues [27]. HEC-LTT and HFF were seeded in μ-slides with 8 wells (ibidi GmbH, Gräfelfing, Germany) at a density of 4 × 10^4^ cells/well. Before seeding HEC-LTT, the wells were covered with 0.1% gelatin (Sigma-Aldrich, St. Louis, MO, USA). The cells were infected with freshly prepared viral supernatant of a late-stage infected HFF culture. After 20 min of infection, the viral supernatant was removed and replaced with cell-specific medium for 5 h and 40 min. HFF and HEC-LTT were fixed by incubation with 80% acetone for 5 min. Antibodies against pp150 and IE86 were used as primary antibodies to stain for viral capsids and infected cells in an indirect immunofluorescence. DAPI solution (Sigma-Aldrich, St. Louis, MO, USA) was added to the wells in order to visualize cell nuclei. Cells were analyzed by a Zeiss Observer Z1 microscope (Zeiss, Oberkochen, Germany) in order to determine expression of IE1 and calculate the proportion of viral capsids at cell nuclei to the viral capsids per total cells.

### 4.6. Antibodies

Following antibodies were used to visualize PML-NB proteins: pAb-PML A301-167A and pAb-PML A301-168A (Bethyl Laboratories, Montgomery, TX, USA), mAb-PML G8 (Santa Cruz Biotechnology, Inc., Dallas, TX, USA), pAb-Sp100 GH3 for WB (kindly provided by H. Will, Hamburg, Germany), pAb-Sp100 B01 (Abnova, Taipeh, Taiwan), mAb-Daxx MCA2143 (Bio-Rad Laboratories, Hercules, CA, USA), mAb-Daxx E94 (Abcam, Cambridge, UK), pAb-ATRX H300 (Santa Cruz Biotechnology, Inc., Dallas, TX, USA), mAb-ATRX 39f (Sigma Aldrich, St. Louis, MO, USA). Following antibodies were used to stain for viral proteins: mAb-IE1 p63-27 [43], pAb-IE2 pHM178 [44], pAb-IE86 for simultaneous detection of IE1 and IE2 [45], mAb-UL44 [46], mAb UL69 [47], pAb-UL84 [44], mAb-MCP 28-4 [48], mAb-pp150 (a kind gift from the Sinzger lab, Ulm, Germany). β-Actin was stained using mAb-β-Actin AC-15 antibody (Sigma Aldrich, St. Louis, MO, USA).

### 4.7. TaqMan Real-Time PCR

Quantitative Real-Time PCR was performed to determine the number of intracellular and released viral genomes of infected HEC-LTT. For this, cells were seeded at a density of 3 × 10^5^ cells/well 6-well plates and, the next day, infected at either a multiplicity of infection (MOI) of 0.05 or a MOI of 0.5. After 90 min of incubation at 37 °C, cells were washed twice with PBS and supplied with heparin-free medium to allow subsequent quantification of viral DNA by qPCR. Viral supernatants from infected cells were harvested five days after infection and incubated with Proteinase K at 56 °C for 1 h. In addition, viral DNA was extracted from infected cells using DNeasy blood and tissue kit (Qiagen, Hilden, Germany) according to the instruction manual. Viral genome copies were then quantified by real-time PCR using an Agilent AriaMx Real-time PCR System together with the corresponding software Agilent Aria 1.5. A sequence in the HCMV gB gene region of viral DNA was amplified using primers 5′CMV_gB and 3′CMV_gB together with a fluorescence-labeled hydrolysis probe CMV_gB_FAM/TAMRA (Table 2). For intracellular DNA samples, cellular Albumin copies were amplified using primers 5′Alb and 3′Alb together with a fluorescence-labeled hydrolysis probe, Alb_FAM/TAMRA (Table 2). For determination of reference C_T_ values (cycle threshold), serial dilutions of the respective standards (10^8^-10^2^ DNA molecules) were examined by PCR reactions in parallel. The 20 μL reaction mix contained 5 μL sample or standard DNA together with 10 μL 2x SsoAdvanced Universal Probes Supermix (Bio-Rad Laboratories, Hercules, CA, USA), 1 μL of each primer (5 μM stock solution), 0.3 μL of probe (10 μM stock solution), and 2.7 μL of H_2_O. The thermal cycling conditions contained an initial step of 3 min at 95 °C followed by 40 amplification cycles (10 s at 95 °C, 30 s 60 °C). The copy numbers of viral genomes and albumin were finally calculated using the sample-specific C_t_ value set into relation to standard serial dilutions.

## 5. Conclusions

In summary, by directly comparing the antiviral activity of PML-NB proteins in HEC-LTT and HFF cells, we found that PML acts as potent inhibitor of HCMV replication in endothelial cells, while Daxx exerts an unexpected pro-viral function. Since we observed that all PML-NB components are expressed in both cell types and localize to nuclear foci, we assume that the cell type-specific behavior arises from differing protein-protein interactions. Finally, our data suggest an important inhibitory role of PML in HCMV infection of endothelial cells and, consequently, for viral dissemination and development of vascular disease.

## Figures and Tables

**Table 1:** siRNA target sequences.

Target Gene	siRNA Sequence
PML	AGATGCAGCTGTATCCAAG
Sp100	GGAAGCACTGTTCAGCGATGT
ATRX	GAGGAAACCTTCAATTGTA
Daxx	GGAGTTGGATCTCTCAGAA
Control	GTGCGTTGCTAGTACCAAC

**Table 2:** Primer and probes for qPCR.

Name	Sequence
5′CMV_gB	CTGCGTGATATGAACGTGAAGG
3′CMV_gB	ACTGCACGTACGAGCTGTTGG
CMV_gB_FAM/TAMRA	CGCCAGGACGCTGCTACTCACGA
5′Alb	GTGAACAGGCGACCATGCT
3′Alb	GCATGGAAGGTGAATGTTTCAG
Alb_FAM/TAMRA	TCAGCTCTGGAAGTCGATGAAACATACGTTC

## Data Availability

Not applicable.
